# Fabrication of ZnO@Ag@Ag_3_PO_4_ Ternary Heterojunction: Superhydrophilic Properties, Antireflection and Photocatalytic Properties

**DOI:** 10.3390/mi11030309

**Published:** 2020-03-15

**Authors:** Huan Huan, Huge Jile, Yijun Tang, Xin Li, Zao Yi, Xiang Gao, Xifang Chen, Jian Chen, Pinghui Wu

**Affiliations:** 1 Joint Laboratory for Extreme Conditions Matter Properties, Southwest University of Science and Technology, Mianyang 621010, China; huanhuan1234567890@yeah.net (H.H.); lixin1010106@yeah.net (X.L.); chenxifang1988@yeah.net (X.C.); chenjian346@yeah.net (J.C.); 2 School of Science, Huzhou University, Huzhou 313000, China; hgjl@zjhu.edu.cn; 3 College of Science, Zhejiang University of Technology, Hangzhou 310023, China; tyj1970@zjut.edu.cn; 4 Research Center for Photonic Technology, Fujian Key Laboratory for Advanced Micro-nano Photonics Technology and Devices & Key Laboratory of Information Functional Material for Fujian Higher Education, Quanzhou Normal University, Quanzhou 362000, China

**Keywords:** ZnO@Ag@Ag_3_PO_4_, nanorods, heterojunction, photogenerated electron, photocatalytic

## Abstract

A ZnO seed layer was formed on the fluorine-doped tin oxide substrate by magnetron sputtering, and then a ZnO nanorod was grown on the ZnO seed layer by a hydrothermal method. Next, we prepared a single-crystal Ag seed layer by magnetron sputtering to form a ZnO@Ag composite heterostructure. Finally, Ag_3_PO_4_ crystals were grown on the Ag seed layer by a stepwise deposition method to obtain a ZnO@Ag@Ag_3_PO_4_ ternary heterojunction. The composite heterostructure of the material has super strong hydrophilicity and can be combined with water-soluble pollutants very well. Besides, it has excellent anti-reflection performance, which can absorb light from all angles. When Ag exists in the heterojunction, it can effectively improve the separation of photo-generated electrons and holes, and improve the photoelectric conversion performance. Based on the above characteristics, this nano-heterostructure can be used in the fields of solar cells, sensors, light-emitting devices, and photocatalysis.

## 1. Introduction

With the rapid development of the global economy, people’s living standards have been greatly improved. At the same time, environmental and energy issues have become increasingly prominent [1,2]. To alleviate these problems, the development of clean and renewable energy has become a current research hotspot. Among these renewable energy sources, solar energy is a cheap, safe, clean, and easily accessible renewable energy source. Making full use of solar energy can solve environmental and energy problems [3,4,5,6,7,8,9,10]. 

ZnO is a semiconductor material with a band gap of 3.37 eV at room temperature [11,12]. Since the 1990s, due to its exciton binding energy of up to 60 meV, thermal stability, high chemical stability, photosensitivity, and catalytic activity, suitable band gap, rich and controllable morphology, environmentally friendly characteristics, and other advantages, it has been widely used in sensors, varistors, and optoelectronic devices [13,14,15,16,17,18,19,20]. Its bandgap is larger than the photon energy (3.10 eV) of visible light at room temperature, resulting in high transmittance in the visible light band, making it an ideal UV light-absorbing material [21]. However, for pure ZnO, the photocatalytic performance is not very good due to the recombination of photo-generated holes (h+) and photo-generated electrons (e^−^). In recent reports, it was pointed out that when heterostructured ZnO nanorods exist, they have stronger photocatalytic performance [22]. Recent studies have shown that when Ag is deposited on ZnO nanorods, Ag acts as a bridge and will inhibit the recombination of photogenerated electrons (e^−^)-h^+^ in ZnO nanorods [23].

Ag_3_PO_4_ has a lower valence band potential, generates photogenerated holes (h^+^), and therefore has a strong oxidation effect [24,25]. It has excellent photocatalytic properties and can degrade organic pollutants in natural light [26]. The photocatalytic performance of Ag_3_PO_4_ crystal under natural light is higher than that of conventional n-doped TiO_2_ [27]. Its photocatalytic performance is limited by the separation of photogenerated charges, transmission efficiency, and ability to capture light. In recent reports, many researchers have selected appropriate metals or semiconductors to combine with target semiconductors to form specific multiple heterostructures [28,29,30,31,32,33,34,35], and this heterostructure can effectively regulate the energy band structure, change the distribution of surface charge, improve the photoelectric performance of the entire material. For example, there are reported a series of metal/oxide semiconductor heterostructures in recent reports, such as fluffy worm-like Ag core nanowires (NWs)-ZnO branched nanorods (ZnO BNRs) and dandelion-like Au core nanoparticles (NPs)-ZnO nanorods (ZnO NRs) heterostructures [36,37], and because of their heterostructure characteristics, they show stronger photocatalytic performance than ZnO nanorods. Similarly, by supporting Ag nanoparticles on the Ag_3_PO_4_ semiconductor to form a heterogeneous photocatalyst, it can effectively improve the absorption efficiency of natural light, broaden the absorption range of the spectrum, effectively separate photo-generated carriers, and use the generated carriers to participate in redox reactions [38].

In this paper, a ZnO@Ag@Ag_3_PO_4_ ternary heterojunction with high photocatalytic performance was prepared by the following steps. Firstly, control the morphology of ZnO and obtain more active sites to participate in the reaction to improve catalytic performance. Secondly, after recombination with silver, due to the different Fermi energy levels, the curved band structure can effectively separate photo-generated carriers and suppress their recombination, while expanding the absorption range of incident light. Finally, the semiconductor-metal interface (Schottky barrier) is formed by the deposition of Ag_3_O_4_, which prevents the recombination of photoinduced electrons(e^−^)-holes (h^+^), and reduces the photo corrosion effect through this interface.

## 2. Experimental

### 2.1. Chemicals and Materials

Ag and ZnO targets with a diameter of 60 mm × 5 mm and purity of 99.99%, zinc nitrate hexahydrate(Zn(NO_3_)_2_·6H_2_O), hexamethylenetetramine(C_6_H_12_N_4_), anhydrous disodium hydrogen phosphate(Na_2_HPO_4_), silver nitrate(AgNO_3_), rhodamine B(C_28_H_31_CIN_2_O_3_), anhydrous ethanol(C_2_H_6_O). All drugs and reagents are analytical pure.

### 2.2. Preparation of ZnO Nanorods

First, the substrate was pre-deposited for 5 minutes at a vacuum of less than 4 × 10^−4^ Pa, an argon flow rate of 40 sscm, and a radio frequency (RF) sputtering power of 60 W. Magnetron sputtering instrument (JGP-560B type high vacuum magnetron sputtering coating system, Shenyang Scientific Instrument Co., Ltd., Chinese Academy of Sciences); Then, the substrate (with an fluorine-doped tin oxide (FTO) film on the surface) was aligned with the center of the ZnO target and sputtered for 5 minutes. ZnO deposited on FTO substrate at a thickness of 10 nm per minute, and the seed layer thickness was about 50 nm. After the sample is cooled, the precursor solution (zinc nitrate hexahydrate and hexamethylenetetramine) is placed at 95 ° C using a hydrothermal method and reacted for 4 hours to grow ZnO nanorods. The mass ratio of Zn(NO_3_)_2_·6H_2_O to C_6_H_12_N_4_ was 1: 1, and the experimental concentrations were 10, 20, 40 and 120 mmol/L, respectively. Finally, the samples were rinsed with deionized water more than three times and dried at room temperature. We label the sample with an experimental concentration of 10 mmol/L as 1#. The sample with an experimental concentration of 20 mmol/L is labeled 2#, the sample with an experimental concentration of 40 mmol/L is labeled 3#, and the sample with an experimental concentration of 120 mmol/L is labeled 4#, and the best-shaped samples were selected by the SEM test for the next step experiment.

### 2.3. Preparation of Ag Layer by Magnetron Sputtering

We use the results obtained in the previous experiment as the substrate for subsequent experiments (3#), and then a metal Ag layer was sputtered by magnetron. The vacuum was 4 × 10^−4^ Pa, and the argon flow rate was 40 sscm, the DC sputtering power was 45 W. We first pre-sputtered for 2 minutes and then officially sputtered each sample for 90 s. After the sputtering experiment was completed, the samples were taken out after cooling for 60 minutes and labeled 5#.

### 2.4. Preparation of Ag_3_PO_4_ by Step Deposition

In this section, AgNO_3_ and Na_2_HPO_4_ solutions were used as the deposition solution [39]. The concentrations of both solutions were 0.02 mol/L, and the volumes were 400 mL and 220 mL, respectively. The number of depositions is a set of variables, and the deposition process was as follows: 5# was placed in the AgNO_3_ solution for 30 min, moved to Na_2_HPO_4_ solution for 5 min, and finally washed with deionized water. All the above steps are regarded as one deposition, and different samples will be obtained for different deposition times. The deposition times of the samples were 1 time, 3 times, 5 times, and 7 times, respectively, and they were marked as 6#, 7#, 8#, and 9# after drying at room temperature.

### 2.5. Photocatalysis Experiment

The Rhodamine B (RhB) was selected as a photocatalytic degradation material and the concentration was 10 ppm. First, 50 mL RhB solution was transferred into a 100 mL beaker at each experiment, and the sample (7.0 × 1.5 cm^2^) was tilted face down on the side of the cup wall. To maintain the adsorption equilibrium, the samples were allowed to stand in RhB solution for 30 min before the experiment. Then FX-300 fiber optic light source (25 mW/cm^2^) was used for subsequent exposure, the light absorption test was performed by liquid UV-visible spectrophotometer (UV-2600A), the content of the degraded organic matter was detected by light absorption. To analyze the degradation process of the reaction solution, the RhB concentration spectrum was recorded once every 30 min by recording the absorption spectrum of RhB.

### 2.6. Experimental Instruments and Sample Characterization

Magnetron sputtering instrument (JGP-560B type high vacuum magnetron sputtering coating system, Shenyang Scientific Instrument Co., Ltd., Shenyang, China) The samples were analyzed by X-ray diffractometry (D/max-1400, Japanese Science, Tokyo Metropolis, Japan). scanning electron microscope (ULTRA55, Zeiss, Oberkochen, Germany) was used to study the surface structure and morphological characteristics of composite samples to analyze the morphology of samples; X-ray photoelectron spectroscopy (XPS) was used to record the chemical states of the elements on a PHI-5702 multi-functional X-ray photoelectron spectrometer (Physical Electronics, Chanhassen, MN, USA); ultraviolet-visible spectrophotometer (UV-2600A, Unico (Shanghai) Instrument Co., Ltd., Shanghai, China) was used to perform light absorption test on the degraded RhB solution; the contact angle tester (DSA30, Kruss, Germany) was used to analyze the hydrophilic and hydrophobic properties of the sample. The test contact angle range is 0°–180°, and the resolution is ±0.01°.

## 3. Results and Analysis

**Figure 1:**
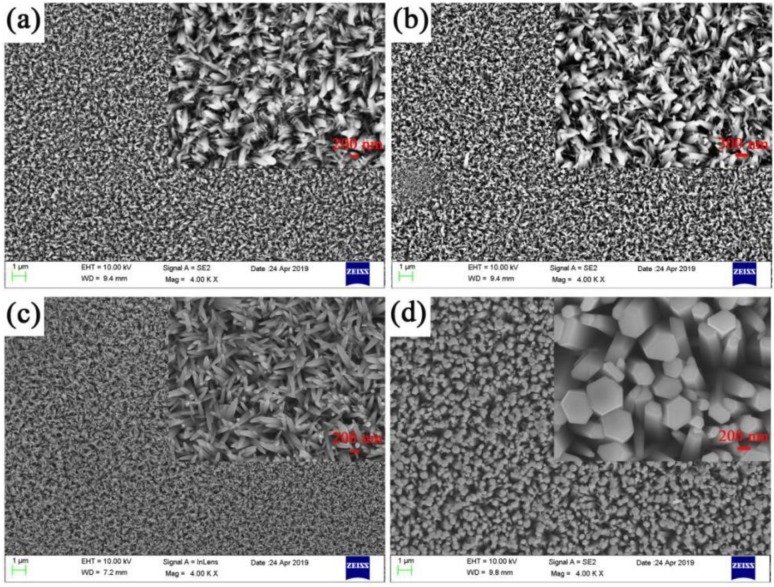
The scanning electron microscopy (SEM) images of ZnO nanorods at different concentrations, size, and uniformity of ZnO nanorods (**a**) 10 mmol/L (sample 1#), (**b**) 20 mmol/L (sample 2#), (**c**) 40 mmol/L (sample 3#), (**d**) 120 mmol/L (sample 4#).

ZnO nanorods are one-dimensional nanomaterials whose structure and morphology have a crucial effect on performance [40]. Figure 1 shows the SEM images of ZnO nanorods under different concentrations. Figure 1a is the SEM image of 1# growth concentration 10 mmol/L. It can be seen that the nanorods in the formed nanorod array are extremely short and unevenly distributed. It was because the concentration of the precursor was too low to crystallization it. Figure 1b shows the SEM image of 2# growth concentration 20 mmol/L. It can be seen that with the increase of the solution concentration, the length and diameter of the nanorods gradually increase, and some hexagonal rod-like structures are gradually formed, but the overall is still uneven. The SEM images of 3# growth concentration 40 mmol/L have been shown in Figure 1c, it was shown that the growth rate and aspect ratio of ZnO nanorods were increased. It meant that the nucleation site increase during hydrothermal was beneficial for the production of ZnO nanorods with high quality and density. Figure 1d is an SEM image of 4# growth concentration 120 mmol/L. It is seen that there is adhesion between the nanorods due to the too high concentration of the precursor solution. Previous research reports have found that the morphology of ZnO has a large effect on performance [40,41,42]. When the precursor concentration was 40 mmol/L, there was no adhesion between the nanorods, and they were evenly distributed.

**Figure 2:**
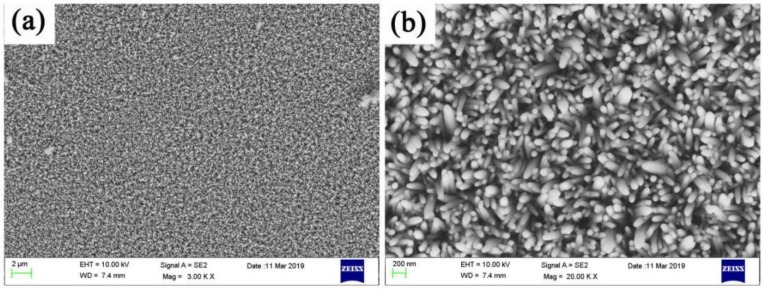
SEM image of Ag sputtered on the surface of ZnO nanorods at low magnification (**a**) and high magnification (**b**) viewing angles, corresponding to sample 5#.

**Figure 3:**
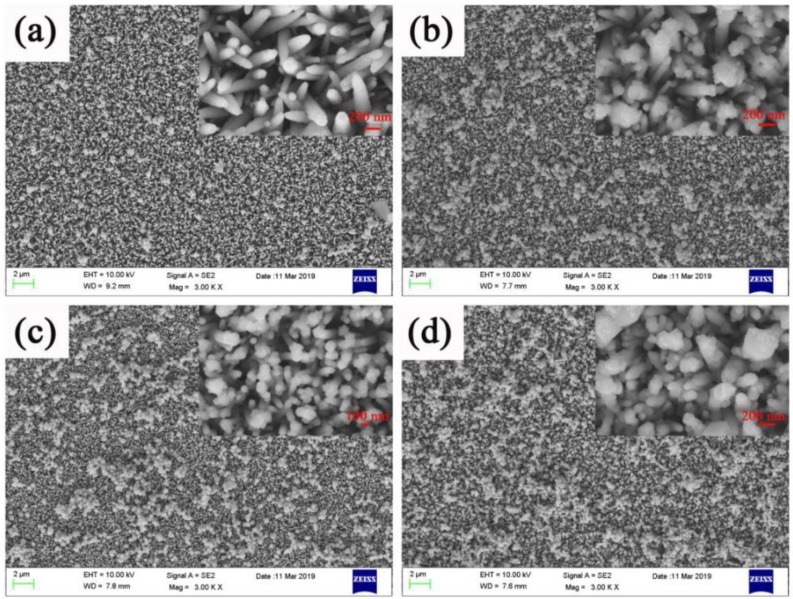
SEM images of ZnO nanorods + Ag + Ag_3_PO_4_: (**a**) Ag_3_PO_4_ deposition once (6#), (**b**) Ag_3_PO_4_ deposition three times (7#), (**c**) Ag_3_PO_4_ deposition five times(8#), (**d**) Ag_3_PO_4_ deposition seven times (9#).

The performance of ZnO nanorods is not ideal, and the photo-generated carrier recombination speed is fast. To suppress the recombination of photogenerated carriers, 3# having the best morphology was selected, and an Ag layer with a thickness of 100 nm was sputtered on the 3# surface. Figure 2 shows the SEM images of 5#. It can be clearly seen that, after the magnetron sputtering of the Ag layer, there was a thin film outline on the ZnO nanorods’ surface. This meant that the Ag layer attached to the ZnO nanorods’ surface successfully. Figure 3 was the SEM images of 6#, 7#, 8#, and 9#. It can be seen that when the number of depositions is one, there are only a small amount of Ag_3_PO_4_ nanoparticles on the surface. When the number of deposition times is three, there are a relatively uniformly distribution of Ag_3_PO_4_ nanoparticles on the surface. As the number of depositions increases to five, many Ag_3_PO_4_ nanoparticles of the same shape and size appear on the surface. When the time of depositions increased to seven, we can see from the picture that the grown Ag_3_PO_4_ nanoparticles are too large and irregular in shape.

**Figure 4:**
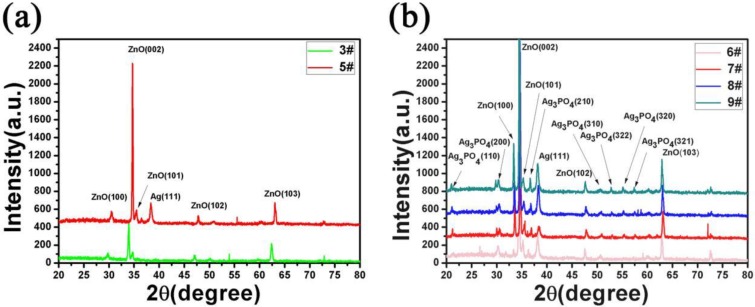
(**a**) The XRD pattern of ZnO nanorods (3#) and ZnO nanorods + Ag (5#), (**b**) the XRD pattern of ZnO nanorods + Ag + Ag_3_PO_4_: Ag_3_PO_4_ deposition once (6#). Ag_3_PO_4_ deposition three times (7#). Ag_3_PO_4_ deposition five times (8#). Ag_3_PO_4_ deposition seven times (9#).

Figure 4a shows the X-Ray Diffraction pattern (XRD) of ZnO nanorods grown on FTO conductive glass (3#) and the XRD test of 5#. It was shown that there were four characteristics of the 3# at 31.75°, 34.39°, 36.23°, and 62.80° which correspond to its (100), (002), (101), and (103) crystal planes of ZnO. Among these diffraction peaks, the (002) diffraction peaks crystal plane was the strongest, indicated that ZnO growth along the (002) crystal plane preferentially, this was because the surface free energy of the (002) crystal plane was lowest [43,44]. For 5#, there was a characteristic diffraction peak of Ag (111) at 38.36° in Figure 4a, this was due to the Ag layer on 5# [45,46]. A shift of diffraction pattern peaks is attributed to the fact that when some silver was sputtered onto the ZnO nano-surface by magnetron sputtering, it entered into the ZnO lattice, which caused the plane reflection X-rays of ZnO in samples 3 and 5 to be different. Figure 4b shows the XRD diffraction patterns of 6#, 7#, 8#, and 9#. It can be seen there were other nine diffraction peaks corresponding to the (110), (200), (210), (102), (310), (322), (320), and (321) crystal planes of Ag_3_PO_4_, respectively [47,48]. Moreover, the intensity of these peaks increases as the number of deposits increases. This is because when the number of deposits is small, there are few Ag_3_PO_4_ nanoparticles, so the characteristic diffraction peaks are not visible. As the number of deposits increases, the amount of Ag_3_PO_4_ nanoparticles increases, and the intensity of diffraction peaks related to Ag_3_PO_4_ gradually increases. These results showed that ZnO@Ag@Ag_3_PO_4_ has been prepared successfully.

**Figure 5:**
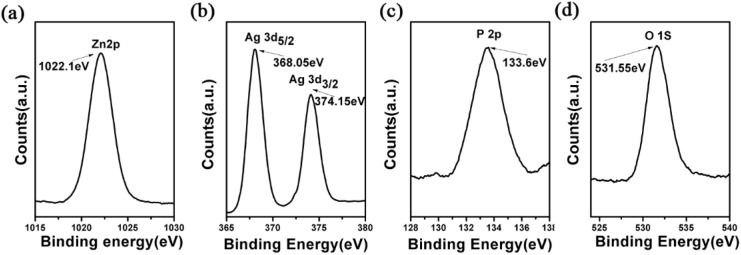
The X-ray photoelectron spectroscopy (XPS) spectra of Zn2p (**a**), Ag3d (**b**), P2P (**c**), and O1S (**d**) in ZnO nanorods + Ag + Ag_3_PO_4_.

X-ray photo-e^−^ spectroscopy (XPS) can provide more info about the chemical state of ZnO@Ag@Ag_3_PO_4_. Figure 5 shows the XPS of 7#. It can be seen there are Zn, Ag, P, and O in 7#. The position of the Zn2p band for ZnO@Ag@Ag_3_PO_4_ (Figure 5a) was at about 1022.1 eV, which meant that the Zn element was the form of Zn^2+^ here [49]. Figure 5b shows the detailed spectra for Ag. It can be seen there were two bands at 368.05 and 374.15 eV which ascribed the Ag 3d5/2 (metallic Ag0 in Ag layer) and Ag 3d_3/2_ (Ag+ in Ag_3_PO_4_), respectively; these results were similar with related reports [50,51]. The Ag 3d5/2 and Ag 3d3/2 bands could be further divided into two different binds at 368.0, 367.5 eV and 374.0, 373.4 eV, respectively [52]. These results indicated there was metallic Ag_0_ in ZnO@Ag@Ag_3_PO_4_. The peak at 531.55 eV was related to lattice oxygen (O 1s) in ZnO and Ag_3_PO_4_ and the peak at 133.6 eV corresponds to the binding energy of phosphorus 2p (P 2p) in Ag_3_PO_4._ From previous reports, there was a minor difference in these data, which was attributed to the ternary heterojunction.

**Figure 6:**
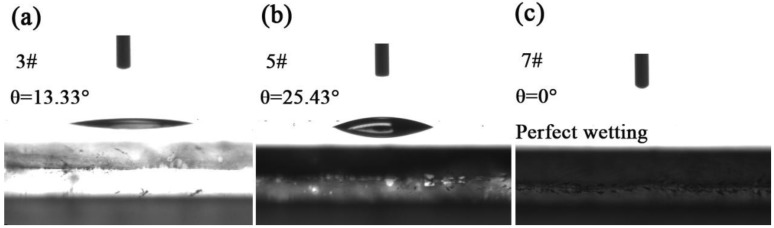
(**a**) Static contact angle of the ZnO nanorods (3#). (**b**) ZnO nanorods + Ag (5#). (**c**) ZnO nanorods + Ag + Ag_3_PO_4_ (deposition three times 7#).

Figure 6a,b show the static contact angles of 3# and 5#, respectively. It was shown that the static contact angle of ZnO was 13.33°. When a 100 nm Ag layer is present on the surface, the static contact angle increases from 13.33° to 25.43°, and the surface hydrophilicity decreases. The reason is that ZnO is a metal oxide, which itself has a certain hydrophilicity. However, Ag is not easily oxidized into a polar metal oxide in the air, and itself has certain hydrophobicity. For 7#, it could be seen from Figure 6c that there was complete wetness and the static contact angle was 0° on the surface—this is because the Ag_3_PO_4_ was an inorganic metal salt and was hydrophilic.

**Figure 7:**
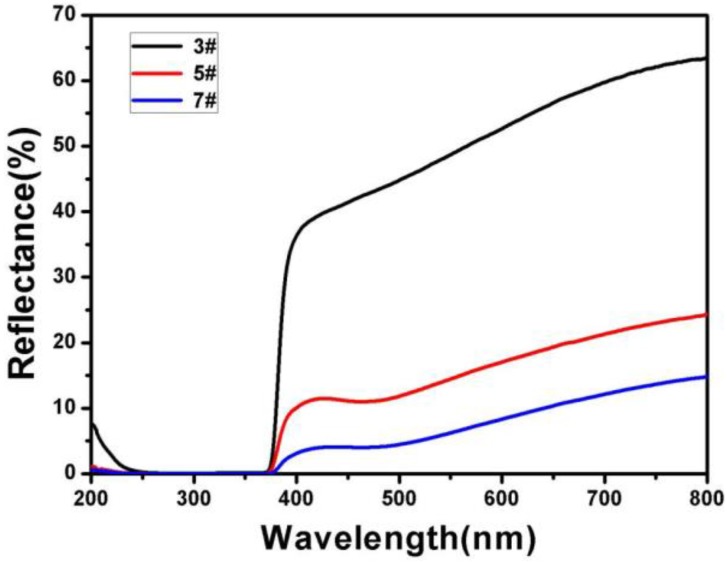
UV-visible reflectance of the sample: (3#) ZnO nanorods. (4#) ZnO nanorods + Ag. (7#) ZnO nanorods + Ag + Ag_3_PO_4_ (deposition three times).

Figure 7 shows the light reflection of 3#, 5#, and 7#. It can be seen that there was reflection from 380 nm to 800 nm of 3#, 5#, and 7#. The reflectance of 3# was approximately 50.94%. The reflectance of 5# was 16.52% and was 34.42% lower than that of ZnO, which meant that the Ag layer in this material was beneficial to light absorption [53,54,55]. The reflectivity of 7# was 8.24%, and was 8.28% lower than 5#, meaning that core-shell composites had a high light absorption.

**Figure 8:**
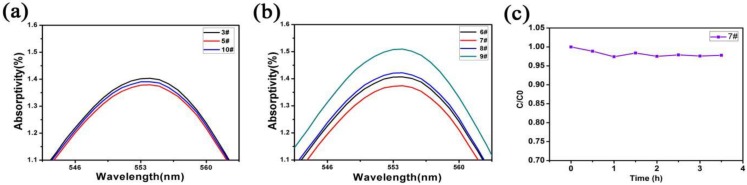
(**a**) The light absorption test of the solution after photocatalysis by 3#, 5#, and 10#; (**b**) the light absorption test of the solution after photocatalysis by 6#, 7#, 8#, and 9#; (**c**) continuous photocatalytic test of 7 # sample.

Figure 8a shows the photocatalytic test results of 3#, 5#, and 10# (5# without Ag layer). The results show that the photocatalytic performance of 5# is higher than that of 3# and 10#, which means that when the surface has an Ag layer, the photocatalytic performance can be improved. The reason is that when there is an Ag layer, the photogenerated e^−^-h^+^ will be separated and utilized due to the potential, thereby reducing its recombination rate. The separation efficiency of the photo-generated carriers was added [56,57,58]. Simultaneously, the Ag layer played an important role in collecting e^−^ by acting as an e^−^ acceptor; it could separate photogenerated carriers effectively and inhibited the recombination of photo-generated carriers produced by ZnO, photocatalytic reduction, and the oxidation reactions of e^−^ and h^+^.

Figure 8b is the photocatalytic test results of 6#, 7#, 8#, and 9#. It can be seen that with the increase of the number of depositions, the photocatalytic performance increases. When the number of depositions is three times (7#), the photocatalytic performance reaches the best. This is because the Ag_3_PO_4_ nanoparticles on the 7# surface are relatively uniformly distributed and are hydrophilic, which can better contact and react with the substances in the solution. Besides, lower reflectivity means more light energy is converted into chemical energy. The 7# had the best photocatalytic properties. Figure 8c is the continuous photocatalytic test of 7#. The results showed that with the increase in degradation time, the prepared samples continued to degrade RhB. The detailed sample number and related characteristics are shown in Table 1.

**Figure 9:**
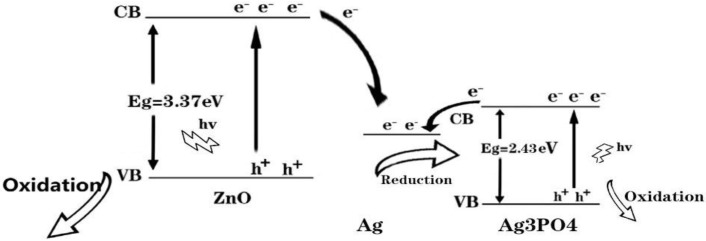
Schematic representation of the ZnO@Ag@Ag_3_PO_4_ heterojunction.

Based on the above results, the reason why the performance of ZnO@Ag@Ag_3_PO_4_ heterostructures was better than Ag_3_PO_4_ nanoparticle and pure ZnO nanorods is discussed in more detail. The Figure 9 illustrated the band structure of ZnO@Ag@Ag_3_PO_4_ heterostructures. When ZnO@Ag@Ag_3_PO_4_ was located at an organic solution and radiated by UV light while photon energy was equal to or higher than the bandgap of ZnO nanorods, the e^−^ in the valence band (VB) could be excited to the conduction band (CB) and the h^+^ would be generated in VB with the same number. As illustrated in Figure 9, the new Fermi energy level of heterostructure was lower than the energy level at the bottom of CB, the photoexcited e^−^ could be moved from ZnO nanorods to Ag through above potential. At the same time, there is an appropriate Fermi level in Ag, which could serve as an excellent e^−^ acceptor for facilitating the transfer of e^−^ from Ag_3_PO_4_ nanoparticle quickly at nature light irradiation [59,60,61]. Thereby, the photoexcited e^−^ could be migrated to Ag quickly, and the photoinduced h^+^ still situated on Ag_3_PO_4_ nanoparticle and ZnO nanorods, which promoted the separation of photoexcited e^−^–h^+^ effectively. As illustrated in Figure 6b, the enriched e^−^ on Ag nanocrystals could promote the multiple-e^−^ reduction reaction effectively, the corresponding reaction formula is as follows [62,63,64], O_2_ + 4H^+^ + 4e^−^ → 2H_2_O.

## 4. Conclusions

In summary, the ZnO@Ag@Ag_3_PO_4_ ternary nano-heterostructure was obtained by magneto-sputtering, hydrothermal method, and deposition successively. The SEM, XRD, and XPS have been tested the morphology, structure, and elements. The study found that the morphology of ZnO nanorods was the best when the concentration of the precursor solution was 40 mmol/L. The presence of Ag in the heterojunction can effectively improve the separation of photoinduced electrons and holes, and therefore has excellent photoelectric conversion properties. The hydrophilicity increased and light reflectance decreased after the deposition of Ag_3_PO_4_.

## Figures and Tables

**Table 1:** Sample table and main features of each sample.

Sample Number	Sample Name	Main Characteristics
1#	ZnO	Array are extremely short and unevenly distributed
2#	ZnO	Nanorods increase in length and diameter, but are not uniform overall
3#	ZnO	Nanorods have the best morphology and size
4#	ZnO	Adhesion between nanorods
5#	ZnO@Ag	UV-visible light reflection is higher than pure ZnO, but hydrophobic
6#	ZnO@Ag@Ag_3_PO_4_	UV-visible light reflection is higher than the ZnO@Ag, but hydrophilic
7#	ZnO@Ag@Ag_3_PO_4_	The photocatalytic performance reaches the best.
8#	ZnO@Ag@Ag_3_PO_4_	UV-visible light reflection is higher than the ZnO@Ag, but hydrophilic
9#	ZnO@Ag@Ag_3_PO_4_	UV-visible light reflection is higher than the ZnO@Ag, but hydrophilic
